# Supplementary material for: Genetic Characterization of Antimicrobial-Resistant Escherichia coli Isolated from a Mixed-Use Watershed in Northeast Georgia, USA
Source: Int J Environ Res Public Health. 2019 Oct 7;16(19):3761. doi: 10.3390/ijerph16193761 (PMC6801870; doi:10.3390/ijerph16193761)
Supplement: Supplementary file 1 [file ijerph-16-03761-s001.pdf]

Table S1. Master file of the 34 antimicrobial resistant *E. coli* isolates from surface water.

Season	Isolate ID	Location	Phylogenetic group	AR profile	AR gene(s)	Replicon Type	integron	MLST
2015 Winter	3 mTEC	MIDO 103	B1	AmpCipNalStrSul TetTri	*bla*TEM, *gyrA* (Val-37→Leu, Ser-83→Leu, Asp-87→Asn), *parC* (Ser-80→Ile), *strA*, *strB*, *sul2*, *tetA*, *dhfr5*	FIB	-	155
	13 mTEC	MIDO 612	F	Tet	*tetA*	FIB, FII	-	117
	15 mTEC	MIDO 616	B1	Tet	*tetA*	FIB	-	156
	25 mTEC	NORO 510	B1	Tet	*tetA*	FII	-	13
	27 mTEC	NORO 605	C	StrSulTet	*strA*, *strB*, *sul2*, *tetB*	FIB, FII	-	10
	13 ECC	MIDO 612	C	Tet	*tetA*	HI1	-	48
	25 ECC	NORO 510	A	Tet	*tetA*, *tetC*	HI1, FIB	-	1172
	27 ECC	NORO 605	B1	Tet	*tetB*	FIB, FIA, FII	-	58
	29 ECC	NORO 609	C	Tet	*tetB*	HI1, FIB, FIC, FII	-	10
2015 Spring	40 ECC	MIDO 504	B1	StrSulTet	*aadA1*, *sul1*, *tetB*	HI1	int1I	196
	65 ECC	MIDO 706	C	Tet	*tetB*	-	-	88
	66 ECC	MIDO 707	C	Nal	*gyrA* (Ser-83 → Leu)	FII	-	43
	95 ECC	NORO 114	E	Tet	*tetB*	-	-	1204
	107 ECC	NORO 503	E	Amp	*bla*TEM	FII	-	69
2015 Summer	161 ECC	NORO 503	B2	Amp	*bla*TEM	FIB, FIA, FII	-	95
	159c mTEC	MIDO 826	E	Tet	*tetA*	-	-	unknown
2015 Fall	164 ECC	BICO 101	B2	AmpNal	*bla*TEM, *gyrA* (Ser-83→Leu, Lys-162→ Gln), *parC* (Lys-247→Glu)	FIB, FII	-	131
	171 ECC	MIDO 505	B1	Tet	*tetA*	FIB	-	297
	192 ECC	MIDO 707	B2	ChlStrSulTet	*flo*, *strA*, *strB*, *sul2*, *tetA*	A/C	-	2604
	205 ECC	MIDO 814	B2	Tet	-	X1	-	372
	207 ECC	MIDO 821	E	Amp	-	-	-	6060
2016 Winter	238 ECC	MIDO 604	E	AmpAziStrSulTetTri	*bla*TEM, *mphA*, *strA*, *strB*, *sul2*, *tetA*, *dhfr5*	P, FII	int1I	69
	255 ECC	NORO 503	B2	Tet	*tetA*	FIB, FIA	-	1170
2016 Spring	264 ECC	BICO 101	A	SulTet	*sul2*, *tetA*	A/C	-	unknown
	274 ECC	MIDO 507	A	AmpStrTet	*bla*TEM, *strA*, *strB*, *tetB*	R, Y	-	216
	279 ECC	MIDO 515	B1	StrSulTet	*strA*, *strB*, *sul2*, *tetB*	-	-	56
	280 ECC	MIDO 601	B2	AmpNal	*bla*TEM, *gyrA* (Ser-83→Leu), *parC* (Ala-192→Gly)	FIB	-	131
	281 ECC	MIDO 604	B1	Tet	*tetA*	Y	-	86
	303 ECC	MIDO 805	B2	AmoAmpFox TioAxoGen	*bla*CMY-2, *bla*TEM-1	I1α, FIB	-	2552

	339 ECC	NORO 527	B1	AmoTet	*tetA*	FIB, FIA	-	58
2016 Summer	353 ECC	MIDO 305	B1	StrTet	*strA*, *strB*, *tetA*	-	-	101
	367 ECC	MIDO 826	B2	AmpTioAxoNal	*bla*$_{CTX-M-15}$, *bla*$_{TEM-1}$, *gyrA* (Ser-83→Leu), *parC* (Lys-247→Glu)	FIB	-	131
2016 Fall	381 ECC	MIDO 103	C	AziSulTetTri	*mphA*, *sul2*, *tetA*, *dhfr12*	I1α, FIB, P, FII	int1I	6826
	382 ECC	MIDO 301	F	Tet	*tetB*	I1α	-	457
